# Enhancing Alzheimer’s Disease and Related Dementia Burden Projections and Policy Targeting in China—Reply

**DOI:** 10.34133/hds.0435

**Published:** 2026-04-13

**Authors:** Xinyi Liu, Luxia Zhang, Yan Li

**Affiliations:** ^1^ School of Public Health, Shanghai Jiao Tong University School of Medicine, Shanghai, China.; ^2^ National Institute of Health Data Science at Peking University, Beijing, China.; ^3^ Renal Division, Department of Medicine, Peking University First Hospital; Peking University Institute of Nephrology; State Key Laboratory of Vascular Homeostasis and Remodeling, Peking University, Beijing, China.; ^4^ Center for Digital Health and Artificial Intelligence, Peking University First Hospital, Beijing, China.; ^5^ Advanced Institute of Information Technology, Peking University, Hangzhou, China.; ^6^ Department of Population Health Science and Policy, Icahn School of Medicine at Mount Sinai, New York, NY, USA.

We thank Dr Chuanqian Liu for the thoughtful and constructive comments on our study projecting the prevalence and economic burden of Alzheimer’s disease and related dementias (ADRDs) across Chinese provinces [1]. We greatly appreciate the positive assessment of our work and the insightful methodological and policy-oriented suggestions, which highlight important directions for strengthening reproducibility, model refinement, and practical relevance in future research [2].

With respect to case identification, ADRD in our study was defined using a survey-based algorithm that required evidence of both cognitive impairment and functional limitation or reported use of medications for memory-related conditions. Cognitive impairment was operationalized as performance at least 1.5 standard deviations below the mean in at least 2 of 3 cognitive domains—time orientation, memory, and arithmetic—with thresholds calculated separately within education strata. This approach follows established conventions in the China Health and Retirement Longitudinal Study (CHARLS) and prior population-based research [3,4], recognizing the strong influence of educational attainment on cognitive test performance and aiming to minimize misclassification. We agree that greater transparency would enhance reproducibility, and in future analyses, we plan to report domain-specific cutoff values by education level as supplementary material.

We also appreciate the suggestion to integrate modifiable risk factors such as physical activity, diet, and air pollution into prevalence projections. These exposures were not included in our baseline models because of incomplete and inconsistently measured lifestyle data across survey waves. Nonetheless, substantial evidence—including findings from the Lancet Commission on dementia prevention—supports their relevance to ADRD risk [5]. CHARLS contains intermittent lifestyle information, and air population indicators can be derived from publicly available provincial monitoring data. We are actively exploring approaches to incorporate these factors into risk-adjusted projection models to examine how changes in behavioral and environmental conditions may influence future ADRD trajectories.

Our projections were designed to reflect a status quo scenario and therefore did not explicitly simulate policy interventions. We agree that dynamic modeling could substantially enhance policy utility by allowing evaluation of strategies such as expansion of long-term care insurance, early dementia screening, and caregiver support [6]. Building on our work, future research could employ microsimulation or system-dynamics approaches to model how policy uptake modifies incidence, care utilization, and costs across provinces, allowing comparative assessment of cost-effectiveness under alternative policy scenarios.

Finally, we acknowledge the importance of disaggregating costs by ADRD subtype and care setting. At present, CHARLS does not provide detailed subtype diagnoses, and linkage to clinical records is limited. Future collaborations with tertiary hospitals may allow integration of diagnostic data to distinguish Alzheimer’s disease, vascular dementia, and other subtypes. Moreover, we are using functional status measures and health-care utilization information in CHARLS to classify broad care settings—such as home-based care, community services, and institutional care where feasible—to estimate setting-specific cost trajectories and better capture heterogeneity in care needs.

We thank Dr Liu again for these valuable suggestions, which will help guide future extensions of this work and strengthen the evidence base for equitable and effective ADRD policy planning across China’s diverse provinces.
